# “NAD‐display”: Ultrahigh‐Throughput in Vitro Screening of NAD(H) Dehydrogenases Using Bead Display and Flow Cytometry

**DOI:** 10.1002/anie.202013486

**Published:** 2021-03-08

**Authors:** Laurens Lindenburg, Florian Hollfelder

**Affiliations:** ^1^ Department of Biochemistry University of Cambridge Tennis Court Road Cambridge CB2 1GA UK; ^2^ Current address: Genmab Uppsalalaan 15 3584 CT Utrecht The Netherlands

**Keywords:** cell-free expression, directed evolution, formate dehydrogenase, saturation library, water-in-oil emulsion droplets

## Abstract

NAD(H)‐utiliing enzymes have been the subject of directed evolution campaigns to improve their function. To enable access to a larger swath of sequence space, we demonstrate the utility of a cell‐free, ultrahigh‐throughput directed evolution platform for dehydrogenases. Microbeads (1.5 million per sample) carrying both variant DNA and an immobilised analogue of NAD^+^ were compartmentalised in water‐in‐oil emulsion droplets, together with cell‐free expression mixture and enzyme substrate, resulting in the recording of the phenotype on each bead. The beads’ phenotype could be read out and sorted for on a flow cytometer by using a highly sensitive fluorescent protein‐based sensor of the NAD^+^:NADH ratio. Integration of this “NAD‐display” approach with our previously described Split & Mix (SpliMLiB) method for generating large site‐saturation libraries allowed straightforward screening of fully balanced site saturation libraries of formate dehydrogenase, with diversities of 2×10^4^. Based on modular design principles of synthetic biology NAD‐display offers access to sophisticated in vitro selections, avoiding complex technology platforms.

## Graphical abstract

A detection system was devised to screen dehydrogenase enzymes (DH) at ultrahigh‐throughput in in vitro droplet compartments. An NAD(H) analogue and a DH DNA library were co‐immobilised on beads. Water‐in‐oil emulsion droplets containing cell‐free expression mix and substrate allow compartmentalised protein production and catalysis. A fluorescent sensor of NAD(H) redox state was loaded onto beads, allowing bulk sorting of 2×10^4^ DH variants in a day by flow cytometry.

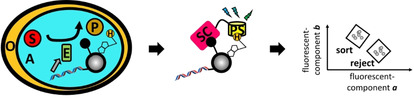

## Introduction

Enzymes play an increasingly important role in the industrial preparation of chemicals, fuelled by sophisticated approaches to improve their properties through mutagenesis and selection of desired variants through screening.^[1]^ The basic steps that make up a directed evolution campaign require a number of choices to be made that can be critically important in determining the successful outcome; DNA library generation (random or (multi)site‐directed), library propagation (in vivo, transformation or in vitro, for example, PCR amplification), protein expression (in cellula or cell‐free), catalysis (in cellula or cell‐free), screening for product formation (directly by chromatography or MS, indirectly using a fluorescent leaving group or a fluorescent *sensor* of product). Arguably, methods for sensitive and selective product detection are key to enabling high throughput screening (e.g. in microfluidic droplet screening systems with capacities >10^6^ per day).[^2^, ^3^] Typically, model substrates with fluorogenic leaving groups[^4^, ^5^] are used for easy optical detection, but their structures (with large, hydrophobic moieties such as fluorescein) often are too different from the substrate of interest, meaning the desired specificity and activity may not be selected for. The more direct the detection of product is, the better will the assay readout propel the screening and selection campaign in the desired direction in sequence space (*“you get what you select for”*). Fluorescent protein‐based sensors, engineered to directly output a robust fluorescent signal in response to small molecule binding, would allow sensitive and specific detection, with a convenient, direct, optical readout. Although such sensors have been developed almost exclusively for the measurement of cell signalling, metabolism and homeostasis,^[6]^ we argue that they present exquisitely sensitive tools with which to carry out high throughput screening.

We exemplify this approach with pyrimidine dinucleotide‐utilising redox enzymes that encompass one sixth of all characterised enzymes^[7]^ and constitute an important target in industrial biocatalysis, for example for the production of optically pure secondary alcohols.[^8^, ^9^] Directed evolution has helped to bring about improved stability, activity and (enantio)selectivity in NAD(P)H‐utilising enzymes.[^10^, ^11^, ^12^, ^13^] Focusing on the redox state of the co‐factor, NAD(H), which many of these enzymes share, we used a best‐in‐class fluorescent protein‐based sensor of NADH, called SoNar, featuring robust, excitation‐ratiometric detection and a large, 1500 % dynamic range^[14]^ to develop an ultrahigh throughput screen. To avoid interference of NAD(H) redox state detection with cellular processes other than the desired transformation, we completely avoid cells by using in vitro transcription & translation (IVTT) to effect expression of NADH dehydrogenase variants, enabling interrogation of large libraries of enzymes without endogenous biological interference. In lieu of the genotype‐phenotype linkage normally provided by cells,^[15]^ we link the redox‐phenotype to the DNA‐encoded genotype on micrometre‐sized paramagnetic beads. We demonstrate our approach, which we call NAD‐display, by ultrahigh throughput selection of a formate dehydrogenase site saturation library, generated using our recently developed SpliMLiB method.^[16]^ NAD‐display is straightforward to implement in molecular biology settings, relying on a modular assembly of functional molecular components. This approach enables ready access to ultrahigh‐throughput screening in droplets,[^2^, ^3^] obviating the need to install microfluidic technologies to create compartments.

## Results and Discussion

### On‐Bead Fluorescent Detection of Immobilised NAD(H) Redox State

**Figure 1:**
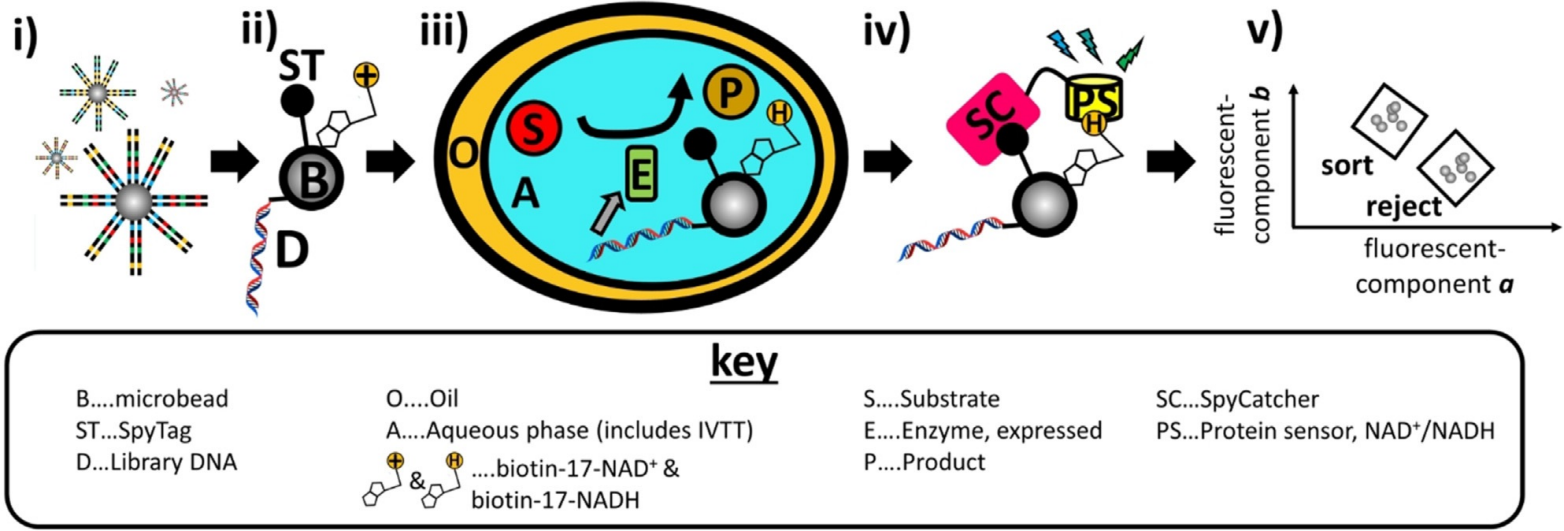
Overview of NAD‐display, using SpliMLiB beads^[16]^ as the library input. (i) First paramagnetic, micrometre‐sized beads, prepared with immobilised SpyTag, as well as functionalities for DNA and biotin binding, are subjected to SpliMLiB,^[16]^ resulting in beads densely coated in “monoclonal” DNA making up a site saturation library of the NAD(H)‐dependent enzyme to be evolved. (ii) After SpliMLiB, beads are furnished with an NAD^+^ co‐factor analogue (biotin‐17‐NAD^+^). [For clarity only a single dsDNA DNA molecule is drawn, although >10^6^ identical DNA molecules exist per bead.^[16]^] (iii) Beads are singly encapsulated in water‐in‐oil emulsion droplets, together with in vitro transcription & translation mixture (IVTT) and the enzymatic substrate. Upon expression of functional dehydrogenase enzyme, catalytic turnover is permanently recorded in the form of reduction of the immobilised co‐factor to NADH. (iv) The emulsion is broken and a fluorescent protein‐based sensor of NAD^+^:NADH is attached to the beads, mediated by isopeptide bond formation between the SpyTag and a SpyCatcher‐sensor protein fusion. (v) Beads are then sorted by flow cytometry, where the specific fluorescent components of the NAD^+^:NADH sensor reveal the functionality of the dehydrogenase encoded by the DNA immobilised on the bead.

NAD‐display uses water‐in‐oil emulsion droplets to generate a phenotype‐genotype linkage by compartmentalization of single genes. In each droplet compartment one protein variant was expressed (by IVTT) from a gene library member attached to a SpliMLiB bead^[16]^ and catalysis took place with an immobilised co‐factor analogue as co‐substrate. Once the substrate is oxidised, the redox state of the co‐factor indicates whether catalysis is complete, which is monitored using an immobilised fluorescent protein‐based sensor. Then beads are screened for product cofactor state, at ultrahigh‐throughput, and selection was achieved using flow cytometric sorting (Figure 1).

**Figure 2:**
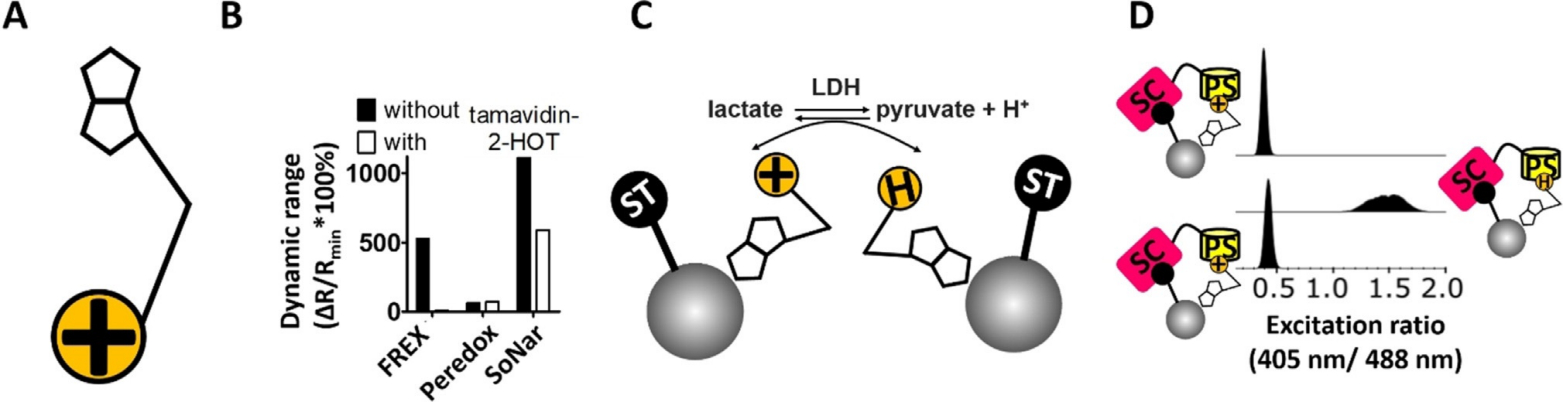
Optimization of NAD‐display bead‐surface sensing and flow cytometry‐based detection of dehydrogenase activity. A) Schematic depiction of the commercially available analogue of NAD^′^ in which biotin and a linker are attached to the adenine end of NAD^+^ (chemical structure shown in Figure S4). B) The in vitro functioning of three different fluorescent protein‐based sensors of NAD(H) redox state was tested with biotin‐17‐NAD^+^ and its reduced form, both in the presence and absence of Tamavidin‐2‐HOT. C) Reduction and re‐oxidation of biotin‐11‐NAD^+^ immobilised on bead. Immobilised co‐factor reduction was brought about by treating beads with LDH and sodium lactate, while the reverse was achieved with LDH and sodium pyruvate. D) Monitoring of bead redox state using SoNar‐SpyCatcher and flow cytometry.

The key design features of the multifunctionalised bead centrepiece of NAD‐display and their practical implementation are:

(1) *Cofactor immobilization*. To immobilise the NAD(H) co‐factor, a biotinylated analogue of NAD^+^ (the commercially available biotin‐17‐NAD^+^, Figure 2 A) was linked to the surface of beads covered with a biotin‐binder. As a biotin‐binding protein Tamavidin‐2‐HOT was chosen and covalently attached to the bead as a fusion protein with the SpyTag^[17]^ in readiness to form an additional attachment point described later in (3). Recombinant expression and purification of Tamavidin‐2‐HOT‐SpyTag fusion protein proved straightforward^[18]^ (unlike streptavidin) allowing us to obtain a high yield of soluble fusion protein (≈80 mg L^−1^).

(2) *Choice of sensor*. A fluorescent protein‐based sensor should report on the NAD^+^:NADH ratio to reliably report the redox‐state of individual beads carrying immobilised co‐factor with an optical signal. Although a number of NADH sensors are on record in the literature,[^19^, ^20^, ^21^] the recently described “SoNar” NADH sensor produces a highly robust signal with a best‐in‐class dynamic range^[14]^ (DR, defined as the maximal change in excitation ratio divided by the minimum excitation ratio^[22]^). We required the probe to bind the NAD(H)‐end of biotin‐17‐NAD^+^ (Figure 2 A), even when the biotin‐end of this analogue was bound to Tamavidin‐2‐HOT. When we tested sensor response in presence and absence of this biotin binder, SoNar was the only probe with high DR for NAD^+^/NADH detection in in the presence of Tamavidin‐2‐HOT (1111 %). FREX^[19]^ suffered a dramatic decline in DR in the presence of Tamavidin‐2‐HOT (8 %), and Peredox,^[20]^ while unaffected by the biotin binder, had a DR that was low in both conditions (71 %), Figure 2 B). SoNar was thus selected for further work.

(3) *SoNar immobilization*. To avoid non‐quantitative labelling of beads due to reversible SoNar:NAD^+^ binding (*K*_d_≈5 μM),^[14]^ the SpyCatcher‐SpyTag system,^[17]^ which allows in situ formation of a post‐translational, covalent isopeptide bond between two protein components, was introduced as a secondary linkage. SpyCatcher was fused to the SoNar C‐terminus, allowing immobilization to the Tamavidin‐2‐HOT‐SpyTag fusion described above (Figure S3). In this way the sensor became covalently attached to the bead‐immobilised biotin‐binder (Figure 2 C).

Together these features should allow each bead carrying one library member gene to be screened for redox reaction turnover by flow cytometric monitoring of cofactor redox state. The ability to reliably distinguish beads carrying either immobilised‐NAD^+^ or its reduced counterpart, was tested with fully assembled beads in a flow cytometric experiment. Beads bearing immobilised NAD^+^ were left either untreated or exposed to lactate dehydrogenase (LDH) and sodium lactate (Figure 2 C). After washing, the beads were labelled with SoNar‐SpyCatcher and profiled by flow cytometry. A clear separation in ratiometric excitation signal, corresponding to their expected redox state, is evident, suggesting that the two samples can be clearly distinguished with excellent DR (277 %, Figure 2 D). To show that the screen also functioned in the opposite direction, potentially allowing valuable reactions such as the reduction of ketones into chiral alcohols to be monitored, the reversibility of the solid‐phase redox reaction was demonstrated (Figure 2 D). Beads were first exposed to LDH and sodium lactate (but not yet SoNar‐SpyCatcher) to achieve oxidation in the forward reaction, were then washed and exposed to LDH and sodium pyruvate (to trigger the reverse reaction, oxidation). The redox state of the NAD^+^/NADH cofactor was monitored by labelling the three bead samples with SoNar‐SpyCatcher. The ratiometric excitation signal of the re‐oxidised beads (Figure 2 D, bottom panel) reflects the starting value (Figure 2 D, top panel) demonstrating the solid‐phase redox reaction was completely reversible.

### NAD‐Display and Catalytic Assays for Formate Dehydrogenase (FDH)

**Figure 3:**
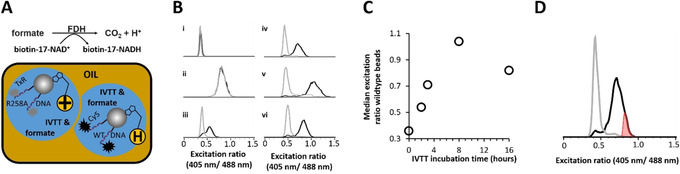
Optimization of NAD‐display incubation time and monitoring of potential for enrichment. A) Schematic representation of IVTT‐expressed wildtype FDH catalysing the reduction of bead‐immobilised biotin‐17‐NAD^+^ using sodium formate as substrate and producing CO_2_. DNA encoding wildtype CaBoFDH was labelled with Cy5 dye, while DNA encoding R258A FDH was labelled with TexasRed (TxR), allowing flow cytometry‐based discrimination of both bead types. Both bead types were mixed and individually encapsulated into water‐in‐oil emulsion droplets in the presence of IVTT and sodium formate. This step resulted in the compartmentalization of single beads in aqueous droplets, together with potential genotype‐dependent reduction of the immobilised co‐factor. B) Excitation ratiometric histograms (normalised to the highest peak) of bead samples measured by flow cytometry. Beads were gated on the basis of TexasRed (grey trace) or Cy5 fluorescence (black trace). Identical aliquots of 1:1 bead mixtures (500 000 beads per sample) were subjected to various conditions during the IVTT and catalysis phase: i) beads exposed to IVTT but not sodium formate; ii) beads exposed to IVTT and sodium formate but without emulsification; iii–vi) beads exposed to IVTT and sodium formate, within an emulsion that was incubated at 25 °C for iii) 2 hours; iv) 3 hours; v) 8 hours; vi) 16 hours. Emulsions (i.e. samples iii–vi*)* were chemically broken after the specified time period, all samples were labelled with SoNar‐SpyCatcher and subjected to flow cytometric analysis. The diagram in vi) shows a clear distinction of positive and negative hits that was gated for selection as shown in panel D. C) Median excitation ratio (405 nm/488 nm, emitting at 520 nm) of beads identified as carrying wildtype CaBoFDH DNA (due to the presence of Cy5) as a function of emulsion IVTT incubation time. D) Determination of the optimal sorting gate (shaded in red) for sample iv) in panel B. The potential n‐fold enrichment was calculated by dividing the ratio of beads functionalised with DNA encoding wildtype CaBoFDH (i.e., positives, black trace) to beads with DNA encoding inactive mutant R258A enzyme (i.e., negatives, grey trace) within the sorting gate (406) by the same ratio within the entire sample (1.3).

With the NAD‐display assay in hand, we next tested its functioning in the context of compartmentalised, in vitro protein expression. We chose to focus on the enzyme formate dehydrogenase from *Candida boidinii*;^[23]^ CaBoFDH is used for biotransformations, where NAD^+^ is typically recycled back to the reduced NADH form using sodium formate as a sacrificial substrate.^[24]^ We confirmed that CaBoFDH was able to accept the unnatural NAD^+^ analogue, consistent with previous reports that many dehydrogenases can accept modifications of NAD(H) at the adenine end of this substrate.[^25^, ^26^, ^27^] However, it should be noted that in the presence of Tamavidin‐2‐HOT, there was a marked reduction in CaBoFDH activity with biotin‐17‐NAD^+^, likely a result of steric hindrance (Figure S6). Nevertheless, activity was still readily detectable in the latter situation and future implementations of NAD‐display may be able to address this lowered activity by synthesizing analogues with longer linkers between the biotin and adenine moiety of biotin‐17‐NAD^+^. DNA encoding wild‐type CaBoFDH and DNA encoding an inactive mutant of CaBoFDH (R258A) were separately loaded on Tamavidin‐2‐HOT‐SpyTag beads, with DNA labelled with two different distinguishable fluorescent dyes (Figure S7B), before biotinylated co‐factor was bound to these beads. Beads were singly encapsulated with IVTT mixture in the droplets of a water‐in‐oil emulsion, together with 25 mM of sodium formate substrate (Figure 3 A). The emulsions were then incubated for several time periods, before they were chemically broken. To reveal the NAD^+^:NADH ratio of each bead, these were loaded with the SoNar‐SpyCatcher sensor and subjected to flow cytometry. Upon addition of the enzyme's substrate, a shift in the excitation ratio (from 0.36 to 0.81, Figure 3 B i) and ii), respectively) indicated successful reduction of bead‐bound NAD^+^, albeit without a phenotypic linkage to genotype, due to the bulk conditions employed during catalysis for sample ii. When beads had been singly encapsulated in a water‐in‐oil emulsion during the expression & catalysis phase, a clear separation of phenotypes could be discerned (Figure 3 B, iii)–vi)). This separation thus also indicates that beads were largely singly encapsulated (consistent with the assumption of a Poisson distribution) as otherwise the phenotype of the beads that had been functionalised with DNA encoding the inactive CaBoFDH mutant would have merged with the phenotype of beads functionalised with DNA encoding active, wildtype CaBoFDH (i.e., as in Figure 3 B, ii)). Incubation of the emulsion sample for 16 hours led to an unexpected reduction in excitation ratio (Figure 3 C). This observation can be explained by a NADH‐oxidising background activity in the IVTT system, becoming apparent only once the rate of reduction slowed down to a sufficiently low level, due to depletion of the sodium formate substrate. Indeed, when a control experiment was carried out to probe directly for background oxidation by IVTT of NADH, this effect was clearly demonstrated (Figure S5). To avoid this reverse background reaction from interfering with the assay and to find the optimal sorting gate, we further exploited the data to calculate the optimal sorting gate that would lead to the highest possible enrichment of wildtype from inactive mutant. As we were able to flow cytometrically establish the genotype of each bead, we could determine that the sorting gate as indicated (Figure 3 D), would lead to an enrichment of 312‐fold. In addition, the fact that all possible events could be accounted for, including true positives and false positives, enabled the positive predictive value^[28]^ of the screen to be calculated: when applying this sorting gate, it was found to be 99.1 %. Even accounting for potential mis‐sorting events caused by a sorting flow cytometer, this established an excellent maximal enrichment. Although this stringent gate does come at the inevitable cost of a relatively high false negative rate (Q1 in Figure S7C), the ability to greatly oversample the library size (see library screen below) will allow many hits to be selected.

### Combining NAD‐Display with SpliMLiB to Test 20 000 CaBoFDH Mutants

**Figure 4:**
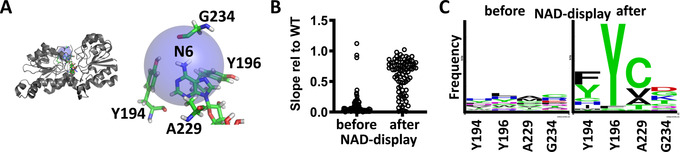
CaBoFDH SpliMLiB library design and screening by NAD‐display. A) On the left, the structure of CaBoFDH (PDB 5DN9) is shown with the targeted sites, Y194, Y196, A229 and G234, depicted in green and the 5 Å radius around the exocyclic nitrogen of adenine shown as a blue transparent sphere. On the right, the targeted residues and adenine are depicted in greater detail. Each of these residues was varied with codons encoding 12 different amino acids (Table S5), resulting in SpliMLiB library size of 20 736. B) Secondary screening of bacterial lysate before (input) and after (sorted output) flow cytometric sorting of beads. Progress curve slopes were normalised to wild‐type CaBoFDH lysate activity measured with 10 mM NAD^+^ and 10 mM sodium formate in a buffer consisting of 20 mM Tris‐HCl (pH 8) and 100 mM NaCl. C) Sequence logo depicting the frequency of amino acids encountered at each position for a total of 36 clones sequenced each for the beads before and after sorting.

FDH is prized in industry for its acceptance of a relatively cheap substrate with which to recycle NAD^+^ back to NADH, as well for its catalysed reaction, in which the escape of the gaseous reaction product (CO_2_) helps to drive the reaction equilibrium forward.^[24]^ However, the CaBoFDH and its homologues suffer from a relatively poor stability^[29]^ and *k*_cat_, while their specificity for NADH has limited their application for recycling of NADPH.[^30^, ^31^] Work by Arnold et al.^[32]^ showed that targeting sites for mutagenesis close to the binding site of the adenine end of NADH led to marked improvements in *k*_cat_ in a diverse set of dehydrogenases.^[32]^ Inspired by this work, we set out to screen a library of four sites simultaneously saturated, using NAD‐display. The sites, Y194, Y196, A229 and G234 were chosen based on their distance (maximally 5 Å) to adenine's exocyclic nitrogen (Figure 4 A).

To generate beads densely coated with DNA encoding single variants of FDH (i.e. monoclonal beads), SpliMLiB libraries were generated. In this recent library approach^[16]^ beads are split into separate tubes, a DNA fragment carrying a different codon at a targeted site is immobilised, and beads are pooled and split again. Solid phase ligation is used to further extend the DNA and, at the same time, for introducing a mutation at another targeted site, with the split & pool process repeated.^[16]^ We limited the number of residues per site to 12, keeping the library to a moderate size of 12^4^=20 736 variants, by limiting replacement in saturation mutagenesis to those amino acids with side chains possessing similar physical and/or chemical properties to the wild‐type amino acid's side chain (Table S5). A detailed, sequence‐level overview of the library construction design is provided (Supplementary Figure S2).

The CaBoFDH SpliMLiB beads (1.5 million) were subjected to selection, using a 4‐hour incubation at 25 °C for the compartmentalised expression & catalysis phase. The incubation time was deliberately set to introduce a stringent selection, as we had previously established apparent saturation at the 8‐hour mark (Figure 3 C). DNA from the sorted beads (Figure S8) and—as a negative control for SpliMLiB‐NAD‐display‐mediated enrichment of functional variants—from beads not subjected to NAD‐display selection was recovered by PCR, cloned into an acceptor vector and bacterially expressed. Enzyme activity was measured in bacterial lysate in a plate reader (Figure 4 B & Table S8 & S9). We can now use the sequence output of this selection in a hotspot analysis that assesses which amino acid substitutions remain functional. The sequences of selected sorted hits (Table S9) displayed a limited number of different amino acids at each position, with most hits displaying activity close to wild‐type. Interestingly, position Y196 was almost completely conserved as the wild‐type amino acid residue, with the very small number of alternative residues found at this position attributable to low‐activity false positives (Table S9). By contrast, position G234 was found not to have been under selective pressure in our screen and we thus conclude that it does not appear to play a significant role, despite being located within 5 Å of the adenine end of NAD^+^. A comparison with the input beads showed that these displayed low activity (Figure 4 B & Table S8) and a near‐random distribution of amino acids at each of the target positions, indicating that the SpliMLiB library was indeed randomised in each position (Figure 4 D). The recovery of clones with wild‐type activity, but different sequences suggests that selections for an activity that is higher than the rest of the library pool are possible, validating the NAD‐display selection workflow.

## Conclusion

We have established a new UHT assay principle, in which beads are equipped with multiple functions—linking genotype and phenotype, carrying the product detection sensor and a reaction cofactor. NAD‐display supports ultrahigh throughput selections of NADH dehydrogenases through flow cytometric sorting of beads displaying immobilised NAD(H) and an NADH fluorescent sensor attached to those same beads. As such, NAD‐display falls under the “display” category of high throughput screening of enzymes, which are more commonly embodied by cell display technologies such as yeast^[33]^ and bacterial display,^[34]^ as well as phage display[^35^, ^36^, ^37^] and retroviral display.^[38]^ In certain cases, it has proven possible to select enzyme activity where both the enzyme and its product remain immobilised on the same surface, allowing genotype‐phenotype linkage without the need for artificial compartmentalization.[^33^, ^36^, ^37^] However, to select enzymes with small‐molecule products, it is typically necessary to create artificial compartments from the start of catalysis up to the point of screening.^[34]^ The advantage that NAD‐display provides is a physical decoupling between the catalysis phase—occurring within artificial compartments—and the screening phase, which is carried out on the bulk, non‐compartmentalised bead population. NAD‐display is thus a user‐friendly approach, as it allows a pause point to be introduced between the catalysis phase and the screening and sorting phase. This is an important advantage, as the flexibility with regard to incubation time is crucial for the level of stringency (by setting a limit on the number of turnovers for variants with poor *k*_cat_), a key parameter in a directed evolution experiment.

To explore sequence space of NAD(P)H dehydrogenases beyond the limitations of plate‐based campaigns, several groups have developed ultrahigh throughput screens. In redox balance screens, the bacterial host cell's native NADH‐consuming pathway is genetically perturbed, such that cells carrying an NADH‐dehydrogenase library variant successfully turning over NADH enjoy a growth advantage.^[39]^ This approach, which has since been adjusted to allow for NADPH‐utilising dehydrogenases,^[40]^ benefits from straightforward selection schemes, but is limited to cell membrane‐permeable and cell growth‐compatible substrates. Similarly, a selection scheme in which a redox‐sensitive transcription factor (TF) produced GFP in response to NADPH‐oxidation by enzyme variants of interest suffered from cell‐to‐cell heterogeneity.^[41]^ The central positioning of NAD(H) and its associated enzymes in basic biochemical pathways renders these live, whole cell‐based screens troublesome. An alternative approach was demonstrated recently where a microfluidic device was employed to optically select droplets of a water‐in‐oil emulsion containing single lysed *E. coli* cells, substrate, NAD^+^ co‐factor and a chemical sensor of NADH.^[11]^ However, the required expertise in microfluidic device creation and sorting remains a significant hurdle to take‐up. NAD‐display, by contrast, simply relies on on‐bead assembly of components that are either commercially available or easily produced as recombinant proteins. Instead of a microfluidic device, a simple filter brings about emulsion droplets. NAD‐display substitutes on‐chip sorting with a flow cytometric sorter, that is, temporary access to a widely used instrument is the only requirement for sorting. NAD‐display thus helps “democratise” UHTS by enabling access to this important technique within biocatalysis for non‐microfluidics‐specialised laboratories, for which there is a pressing need.^[42]^

Unlike most other examples listed above, NAD‐display is an entirely in vitro platform, using cell‐free expression, meaning it is i) time‐saving, as bacterial transformation steps to generate the screening library (typically taking days) are skipped; ii) insensitive to potential cell‐toxicity problems associated with either enzymes themselves or their substrates & products. Furthermore, we showed here how NAD‐display could be directly integrated with SpliMLiB, allowing fully non‐degenerate, multi‐site saturation. NAD‐display could therefore also fit within directed evolution approaches such as ISM and CASTing,^[43]^ as well as approaches where sequence fragments from homologous proteins are shuffled.^[44]^

More generally, the design of the display construct is based on the ambition of synthetic biology to create bespoke complex functionality by assembly of simple building blocks. The modular design principles of NAD‐display may become a blueprint for a general assay principle based on two requirements, that is, that enzymatic products can be readily immobilised and a fluorescent sensor is available. For applications beyond NADH dehydrogenases, NADPH‐dependent enzymes may be monitored by iNAP^[45]^ or the redox cofactor may be conceptually replaced by ATP, using sensors such as ATeam^[46]^ or iATPSnFRs^[47]^ providing access to ultrahigh throughput screening of ATPases/ATP synthetases or other enzymes coupled to this cofactor. As more sensors become available, a variety of functional manifolds in analogy to NAD‐display can be constructed, enabling UHT campaigns to identify and improve enzymes catalysing a much wider range of reaction than currently possible.

## Conflict of interest

The authors declare no conflict of interest.

## Supporting information

As a service to our authors and readers, this journal provides supporting information supplied by the authors. Such materials are peer reviewed and may be re‐organized for online delivery, but are not copy‐edited or typeset. Technical support issues arising from supporting information (other than missing files) should be addressed to the authors.

Supplementary
