# Supplementary material for: “NAD‐display”: Ultrahigh‐Throughput in Vitro Screening of NAD(H) Dehydrogenases Using Bead Display and Flow Cytometry
Source: Angew Chem Int Ed Engl. 2021 Mar 8;60(16):9015–21. doi: 10.1002/anie.202013486 (PMC8048591; doi:10.1002/anie.202013486)
Supplement: Supplementary file 1 — Supplementary [file ANIE-60-9015-s001.pdf]

Angewandte Chemie
Eine Zeitschrift der Gesellschaft Deutscher Chemiker

Supporting Information

“NAD-display”: Ultrahigh-Throughput in Vitro Screening of NAD(H) Dehydrogenases Using Bead Display and Flow Cytometry

*Laurens Lindenburg and Florian Hollfelder**

anie_202013486_sm_miscellaneous_information.pdf

Contents

Methods 2

Reagents 2

Generation of plasmids used in this study 2

Large scale protein expression and purification 2

Testing DR of different NAD(H) sensors 3

Redox reactions on bead-immobilized NAD(H) 3

SoNar-SpyCatcher coupling to SpyTag on beads 3

Bead flow cytometry 3

Immobilization of DNA on NAD^+ display beads 4

Bead emulsion IVTT & de-emulsification 4

SpliMLiB process 4

Secondary screening of flow cytometrically-sorted hits 4

Supplemental text 5

1. Formation of SoNar-SpyCatcher-SpyTag-Tamavidin-2-HOT complex in solution 5

Figures & Tables 5

References 19

Figure S1: Partial DNA and amino acid sequences of constructs used in this study 8
Figure S2. Sequence-level overview of fragment preparation 10
Figure S3: Preparation of post-translationally fused protein complex 12
Figure S4: Biotin-17-NAD structure 12
Figure S5: Demonstration of background oxidation activity in IVTT mixture 13
Figure S6: Activity of CaBoFDH with biotin-17-NAD^+ substrate 13
Figure S7: Flow cytometry gating scheme and NAD-display assay's positive predictive value 14
Figure S8: FACS sorting histogram 14

Table S1. Oligonucleotides used in this study for non-SpliMLiB cloning 8
Table S2. Common oligonucleotides used to generate DNA for SpliMLiB. 8
Table S3. Oligonucleotides for construction of the CaBoFDH SpliMLiB library 8
Table S4. Codons for site saturation in the SpliMLiB library. 9
Table S5: Amino acids targeted at each position 9
Table S6 PCR amplicons used in this study 9
Table S7. Oligonucleotide duplex used for CaBoFDH SpliMLiB library generation 9
Table S8: CaBoFDH SpliMLiB input library clones' activity 15
Table S9: CaBoFDH SpliMLiB NAD-display screened clones' activity 17

Methods

Reagents

6-Biotin-17-NAD^+ was from BIOLOG Life Science Institute (Bremen, Germany). COOH-functionalized 5 µm diameter paramagnetic silica beads were from Microparticles GmbH (Berlin, Germany). Fluorinated surfactant RAN was from RAN Biotechnologies (Beverly, MA), HFE-7500 oil was from 3M.

Generation of plasmids used in this study

The protein expression construct pET28a-Tamavidin-2-HOT-SpyTag has been described previously (Huovinen *et al.* 2020, submitted) The construct for FREX was ordered as synthetic gene, amplified with oligonucleotides FREXtoPOP_F and FREXtoPOP_R (Table S1) to give SpeI and NotI sites at either end, allowing the construct to be cloned into vector pOP3TB (kind gift from M. Hyvönen, http://hyvonen.bioc.cam.ac.uk/pOP-vectors/) that had been cut with SpeI and NotI. pRSetB-Peredox, encoding the Peredox sensor, was a gift from Gary Yellen (Addgene plasmid # 73806).[1] SoNar was ordered as a synthetic gene (GeneArt, ThermoFisher) and cloned into pHAT-Avi, a pHAT plasmid [2] derivative with N-terminal Avi-Tag, using NcoI and NotI restriction sites. To generate the construct pET28a-SoNar-SpyCatcher (Figure S1A), three different PCR amplicons were first prepared. A vector backbone PCR amplicon was prepared using pET28a (Novagen) as template and the oligonucleotides SpyC_pETTerm_F and Thrombin_SoNar_R. A SoNar-encoding PCR amplicon was prepared using pHAT-Avi-SoNar as template, together with the oligonucleotides Thrombin_SoNar_F and SoNar_NotI_SpyC_R. A SpyCatcher-encoding PCR amplicon was prepared using a synthetic gene (GeneArt, ThermoFisher) as template, together with the oligonucleotides SoNar_NotI_SpyC_F and SpyC_pETTerm_R. Oligonucleotide sequences are listed in Table S1. All three PCR reactions were carried out in a 50 µL Phusion DNA polymerase (NEB) reaction, using 1xbuffer HF, 0.5 µM of either oligonucleotide, 0.2 mM dNTPs, with a thermocycling program consisting of 98 °C (30 seconds), 30 cycles of [98 °C (15 seconds), 72 °C (3 minutes)], 10 minutes 72 °C. The three fragments were combined to form a plasmid using Circular Polymerase Extension Cloning (CPEC) [3]: 100 ng of vector backbone PCR amplicon was combined with equimolar amounts of the other two amplicons, in a 50 µL Phusion Polymerase (NEB) reaction, not supplemented with oligonucleotides, with a thermocycling program consisting of 98 °C (30 seconds), 30 cycles of [98 °C (15 seconds), 72 °C (3 minutes)], 10 minutes 72 °C. Methylated plasmid templates were removed by incubating the reaction mixture with 20 units DpnI (NEB) for 10 minutes at 37 °C, before the preparation was transformed into 20 µL of chemically competent *E. coli* (Alpha-Select Silver Efficiency, Bioline). The cloning of pIVEX-CaBoFDH (Figure S1B) was described previously.[4] For bacterial expression of CaBoFDH, the construct was cloned from pIVEX into pET28a (Figure S1C) using NdeI and BamHI. The inactivating mutation R258A was introduced by site directed mutagenesis of pIVEX-CaBoFDH following a previously described protocol [5] using oligonucleotides R258A_F and R258A_R (Table S1). To allow sub-cloning of DNA recovered from flow cytometrically sorted beads, an Eco31I-containing acceptor vector, pET28a-CaBoFDH_BsaI*2 (Figure S1D), was constructed by the site directed mutagenesis of pET28a-CaBoFDH using oligonucleotides BsaI_Vec_SDM_F and BsaI_Vec_SDM_R (Table S1). DNA and amino acid sequences for the protein-of-interest coding sequences in pET28a-SoNar-SpyCatcher, pIVEX-CaBoFDH, pET28a-CaBoFDH and pET28a-CaBoFDH_BsaI*2 are provided in the Supplementary Information.

Large scale protein expression and purification

Large-scale expression of proteins (Tamavidin-2-HOT, Tamavidin-2-HOT-SpyTag, FREX, Peredox, SoNar and SoNar-SpyCatcher) was carried out in *E. coli* BL21(DE3) in 0.5 litre of LB, inoculated with overnight pre-cultures. The 0.5 litre cultures were grown to an OD_{600} of 0.5, at 37 °C in a shaking incubator, at which point expression was induced by adding IPTG to a final concentration of 100 µM and incubating cells at 25 °C, shaking overnight. Cells were lysed using 10 mL of 1x BugBuster with 1 µL Benzonase (Novagen) in NiNTA buffer (20 mM Tris/HCl, pH 8; 500 mM NaCl) with 5 mM imidazole. Lysate, clarified through centrifugation, was applied to NiNTA resin (1 mL bed volume), washed with NiNTA buffer (20 column volumes containing 5 mM imidazole, then 20 column volumes with 30 mM imidazole). Proteins were eluted using 2.5 mL NiNTA buffer with 500 mM imidazole. This buffer was exchanged to 50 mM Tris-Cl (pH 8), 100 mM NaCl using a

PD-10 desalting column (GE). Protein purity was confirmed by SDS-PAGE and proteins were frozen at -80 °C. SoNar-SpyCatcher was further purified using a size exclusion column (16/60 Superdex 200, GE) as this treatment was found to improve its apparent DR, possibly by removing tightly bound NADH: the SoNar absorbance ratio (420/500 nm) was 1.6 before and 0.7 after SEC purification, consistent with a lower amount of contaminating NADH in the SEC-purified preparation.

Testing DR of different NAD(H) sensors

FREX, SoNar and Peredox proteins were measured at 0.625 µM in a buffer of 100 mM Tris-Cl (pH 8), 100 mM NaCl and 80 µM biotin-17-NAD$^+$, in a fluorescence plate reader (Tecan Infinite 200Pro). Fluorescence excitation ratios for FREX and SoNar (emission for both set to 550 nm) were obtained using 400 and 490 nm (FREX) and 420 and 500 nm (SoNar) excitation. Peredox was measured by excitation at 410 nm and emission set to 510 nm, with signal normalized by its fused mCherry domain (excitation 550 nm, emission 618 nm). To reduce biotin-17-NAD$^+$, LDH (0.1 U of chicken muscle LDH, Specialty Assays Inc) and 25 mM sodium lactate was added. Tamavadin-2-HOT protein (i.e. not fused to SpyTag) was added to a final concentration of 93 µM.). Dynamic range (DR) was defined as,

$$DR = \frac{\Delta R}{R_{min}} \times 100\% \quad (1)$$

, where ΔR is the difference between the fluorescence ratio observed before and after enzymatic reduction of biotin-17-NAD$^+$ and R_{min} is the lower of the two fluorescence ratios observed.

Redox reactions on bead-immobilized NAD(H)

Superparamagnetic silica beads (∅ 5 µm) coated with Tamavidin-2-HOT-SpyTag functionalized with azide were prepared as described previously.[4] To immobilize the NAD$^+$ analogue (6-Biotin-17-NAD$^+$), beads were incubated in 35 µL PBS (with 0.05% (v/v) Tween-20) with 50 µM NAD$^+$ analogue, for 30 minutes at 22 °C, shaking at 1200 RPM. Beads were then washed three times with PBS with 0.05% (v/v) Tween-20. To reduce immobilized NAD$^+$ using LDH, beads were incubated with 0.1 U of LDH and 250 mM sodium lactate, in 100 µL, for 30 minutes at 22 °C, while shaking, before being washed three times with PBS with 0.05% (v/v) Tween-20. To oxidize immobilized NADH, the same procedure was carried out, except 25 mM sodium pyruvate was used instead of sodium lactate. DR was calculated with equation (1), using the median ratios of the flow cytometrically analyzed bead populations (see below) to determine the ΔR and R_{min} parameters.

SoNar-SpyCatcher coupling to SpyTag on beads

Beads were incubated with SoNar-SpyCatcher (17 µM in PBS with 0.05% Tween-20) for 20 minutes at 22 °C on a shaking incubator (1200 RPM). Beads were washed three times with PBS with 0.05% Tween-20.

Bead flow cytometry

Flow cytometric analysis was carried out on a BD LSR-Fortessa system equipped with a 405 nm excitation laser coupled to a 525/50 BP emission filter, a 488 nm laser coupled to a 530/30 BP filter, a 561 nm laser coupled to a 610/20 BP filter and a 640 nm laser coupled to a 670/14 BP filter. The first two of these excitation/emission, laser/filter pairs were used to measure the SoNar fluorescence excitation ratio, while the latter two were used to detect the DNA-coupled fluorophores TexasRed and Cy5, respectively. Data analysis was performed by FlowJo software (BD).

The positive predictive value of the NAD-display screen was calculated by dividing the number of true positive events (Figure S7, Q2) by the sum of the true positives (Q2) and false positives (Q3)[6].

To depict the excitation ratio in histogram format, a new parameter was derived through division of the 405 nm excitation /525/50 nm BP emission parameter by the 488 nm excitation /530/30 nm BP emission parameter. Flow cytometric sorting was performed by using a FACSAria Fusion system (BD), using lasers and emission filter with similar spectral properties to the Fortessa.

Note that for NAD-display, it is essential to use a flow cytometry system where events are successively excited by different lasers orientated in a parallel setup as the event moves through space so that each interrogation point is

associated with its own collection path. A colinear system (e.g., as found in the Sony SH800S flow cytometric sorter), where multiple lasers are focused on a single spot, will not allow excitation ratiometric measurements.

Immobilization of DNA on NAD^+ display beads

DNA that was to be immobilized on the beads was prepared by carrying out PCR using as one of the oligonucleotides T7t-DBCO (Table S2), such that the PCR amplicon carried a DBCO function at one of its 5'-ends (Table S6). DBCO-functionalized DNA (either control constructs or SpliMLiB initiator fragments) was coupled to these beads by a copper-free click reaction,[7] in the presence of crowding reagents (Dynabeads kilobaseBINDER kit (KBBK, ThermoFisher Scientific). Beads were washed once in 40 µL bind solution (KBBK), then resuspended in 40 µL of the same before DBCO-functionalized DNA (40 µL, in MiliQ water with 0.02% Tween-20 (v/v) was added. The reaction was incubated at 37 °C on a shaking platform (1200 RPM) for 1 hour. Beads were washed once in wash solution (KBBK), then three times with PBST (0.05%). DNA was added in a ratio of approximately 5 million molecules of DNA per bead, in a total click reaction coupling volume of 80 µL, with 1 to 2 million beads, equating to DNA concentrations of approximately 0.1 µM.

Bead emulsion IVTT & de-emulsification

Beads (1-3 million) were resuspended in IVTT mix (PURExpress, NEB; 12.5 µL consisting of 5 uL solution A, 3.75 uL solution B, 0.5 uL RNase inhibitor murine (NEB), 2 uL of 156.25 mM sodium formate and 1.25 uL water). Immediately upon resuspension, the bead/IVTT mixture was transformed into a water-in-oil emulsion by passing the bead/IVTT mixture repeatedly through a 20 µm filter device until the emulsion appeared homogeneous (i.e. lacking in large, visible droplets of aqueous phase), as previously described [8,9]. After incubation of the emulsion at 25 °C, the emulsion was broken by the addition of 100 µL PBS with 0.05% Tween-20 and 18 µL of perfluorooctanol (PFO, Alfa Aesar), followed by vigorous mixing by pipetting. The top aqueous layer was pipetted to a collection tube on a magnet and another 100 µL of PBS with 0.05% (v/v) Tween-20 and 18 uL of PFO was pipetted to extract any remaining beads from the oil. Beads were washed three times with PBS with 0.05% (v/v) Tween-20.

SpliMLiB process

12 different DNA fragments per targeted position in CaBoFDH were prepared. PCR products (Table S6) and oligonucleotide duplexes (Table S7) were prepared as previously described[4] and subjected to appropriate restriction enzyme digestion in solution. These were then assembled onto the beads, by successive ligations, using the split & mix methodology to maintain bead 'monoclonality'. A detailed, sequence-level overview of the SpliMLiB process for the preparation of the CaBoFDH library is provided in Figure S2.

Secondary screening of flow cytometrically-sorted hits

To re-screen hits obtained from flow cytometric sorting (and input library variants), the DNA on sorted beads (and input library variant beads) was amplified by PCR. The PCR consisted of 0.5 uM each of primers BsaI_Lib_F and BsaI_Lib_R (Table S1), 1x Phusion HF buffer, 0.2 mM dNTPs and 2 units Phusion polymerase (NEB), in a 100 µL reaction. The PCR cycling program consisted of: 30s, 98 °C, then 30 rounds of 98 °C 10 seconds and 72 °C for 20 seconds, followed by a single 72 °C step for 5 minutes. PCR amplicons (40 µL) were purified by SPRI[4], using 120 µL SPRI slurry. Golden Gate cloning[10] was used to insert the Eco31I-flanked PCR products into the pET28a-CaBoFDH_BsaI*2 acceptor vector: 0.6 ng insert and100 ng vector were combined with T4 DNA ligase (100 units; NEB) in T4 DNA ligase buffer (1x; NEB) and 0.25 µL Eco31I (FastDigest; ThermoFisher) , in a total volume of 5 µL. The reaction was run in a cycle that alternated between 2 minutes at 37 °C and 2 minutes 16 °C for a total of 30 cycles, before heat inactivation of both enzymes at 80 °C for 10 minutes. Then any remaining Eco31I sites were destroyed by addition of a supplemental 0.25 µL Eco31I, followed by incubation at 37 °C for 30 minutes, transformation to *E. coli* and plating on LB agar plates with 50 µg/mL kanamycin. We found that transformation of 20 µL chemically competent *E. coli* cells (alphaSelect Silver Efficiency, Bioline) with 2 µL of the Golden Gate reaction resulted in the appearance of hundreds of colonies with little to no

background: (less than 5 colonies appeared in a Golden Gate reaction that contained no insert). The resulting colonies were scraped off the plate (by adding 5 mL LB and resuspending cells with an L-shaped spreader) and used for direct plasmid DNA extraction, with the GeneJET Plasmid Miniprep Kit (ThermoFisher Scientific) before plasmid DNA was transformed into *E. coli* BL21(DE3) cells, which were plated on LB agar with 50 µg/mL kanamycin. BL21(DE3) colonies were picked and used to inoculate 96-deep well plates containing 400 µL LB with 50 µg/mL kanamycin per well. After overnight growth of these plates at 37 °C with shaking and shielded by a Breathe-Easy sealing membrane (Sigma-Aldrich), glycerol stocks of the deep-well plates were prepared by transferring 100 µL of overnight culture per well to 50 µL of 50% (v/v) glycerol (in LB, with 50 µg/mL kanamycin) in a standard microtitre plate. The glycerol stock plates were frozen and later used to inoculate a fresh 96-deep well plate, with wells each containing 400 µL LB with 50 µg/mL kanamycin. The following day, after incubation of the deep-well plate at 37 °C shaking overnight, 4 µL from this deep-well plate was transferred to a fresh deep-well plate (with 400 µL LB and 50 µg/mL kanamycin per well). This plate was incubated at 37 °C until the OD_{600} of the cultures was about 0.5, at which point IPTG was added to a final concentration of 100 µM and the plate was incubated at 25 °C overnight, shaking. The following day, the OD_{600} prior to cell harvesting was measured (to aid in later growth-dependent normalization of enzyme activity in lysate). Cells were harvested by centrifugation and cells were lysed by resuspension of the cell pellet (by vortexing) in 200 µL of 1xBugBuster (Novagen) with 5 units benzonase (Novagen) in a buffer consisting of 50 mM Tris-Cl (pH 8) and 100 mM NaCl. The lysate was clarified by centrifugation of the plates at (4000 rpm) for 45 minutes. Then 20 µL of lysate was carefully pipetted off from the top of the lysate (to avoid disturbing the pelleted insoluble material in the plate) and transferred to a standard microtitre plate containing substrate solution for a final concentration of 10 mM NAD^+, 10 mM sodium formate, 50 mM Tris-Cl (pH 8) and 100 mM NaCl. NADH formation over time was monitored by the increase in absorbance at 340 nm using a Spectramax M2 spectrophotometer. Activity slopes were determined using Spectramax software and normalized for the OD_{600} of the bacterial culture giving rise to the lysate, and further normalized to the average of three wild type control wells included in each plate.

Supplemental text

1. Formation of SoNar-SpyCatcher-SpyTag-Tamavidin-2-HOT complex in solution

Tamavidin-2-HOT-SpyTag and SoNar-SpyCatcher (Figure S1A) were expressed in *E. coli* BL21(DE3) and could be purified in good yield (ca 20 mg/litre *E. coli* culture). To bring about the post-translational fusion, Tamavidin-2-HOT-SpyTag (21 nmol, calculated as monomers) and SoNar-SpyCatcher (81 nmol) proteins were mixed in a total volume of 1.15 mL, and incubated overnight at room temperature. The mixture was loaded on a SEC column and the elution trace showed a clear early peak, distinct from the peak obtained with Tamavidin-2-HOT-SpyTag (76 kDa) or SoNar-SpyCatcher (53 kDa) alone (Figure S3B). SDS-PAGE analysis revealed this complex-peak to correspond to the expected molecular weight of a tetramer of SoNar-SpyCatcher-SpyTag-Tamavidin-2-HOT (290 kDa, Figure S3C).

Figures & Tables

A pET28a-SoNar-SpyCatcher

```
atgggcagcagccatcatcatcatcatcacagcagcggcctggtgccgcgcggcagcggt
 M  G  S  S  H  H  H  H  H  H  S  S  G  L  V  P  R  G  S  G
aatcgtaaatggggtctgtgtattgttggtatgggtcgtctgggtagcgcactggcagat
 N  R  K  W  G  L  C  I  V  G  M  G  R  L  G  S  A  L  A  D
tatccgggttttggtgaatcatttgaactgcgtggtttttttgatgtggatccggaaaaa
 Y  P  G  F  G  E  S  F  E  L  R  G  F  F  D  V  D  P  E  K
gttggtcgtccggttcgtggtggtgttattgaacatgttgatctgctgccgcagcgtgtt
 V  G  R  P  V  R  G  G  V  I  E  H  V  D  L  L  P  Q  R  V
ccgggtcgtattgaaattgcactgctgaccgttccgcgtgaagcagcacagaaagcagcc
 P  G  R  I  E  I  A  L  L  T  V  P  R  E  A  A  Q  K  A  A
gatctgctggttgcagcaggtattaaaggtattctgaattttgcaccggttgttctggaa
```

```
 D  L  L  V  A  A  G  I  K  G  I  L  N  F  A  P  V  V  L  E
gttccgaaagaagttgcagttgaaaacgttgattttagcgcaggctataatagcgataac
 V  P  K  E  V  A  V  E  N  V  D  F  S  A  G  Y  N  S  D  N
gtgtatattatggccgataaacagaaaaacggcattaaagccaacttcaaaattcgccat
 V  Y  I  M  A  D  K  Q  K  N  G  I  K  A  N  F  K  I  R  H
aatgtggaagatggtagcgttcagctggcagatcattatcagcagaataccccgattggt
 N  V  E  D  G  S  V  Q  L  A  D  H  Y  Q  Q  N  T  P  I  G
gatggtccggttctgctgccggataatcattatctgagctttcagagcgttctgagcaaa
 D  G  P  V  L  L  P  D  N  H  Y  L  S  F  Q  S  V  L  S  K
gatccgaatgaaaaacgtgatcacatggtgctgctggaatttgttaccgcagccggtatt
 D  P  N  E  K  R  D  H  M  V  L  L  E  F  V  T  A  A  G  I
accctgggtatggatgaactgtataacgttgatggtggtagcggtggcaccggtagcaaa
 T  L  G  M  D  E  L  Y  N  V  D  G  G  S  G  G  T  G  S  K
ggtgaagaactgtttaccggtgttgttccgattctggttgaactggatggtgatgttaat
 G  E  E  L  F  T  G  V  V  P  I  L  V  E  L  D  G  D  V  N
ggccacaaattttcagttagcggtgaaggtgaaggtgatgcaacctatggtaaactgacc
 G  H  K  F  S  V  S  G  E  G  E  G  D  A  T  Y  G  K  L  T
ctgaaactgatttgtaccaccggtaaactgccggttccgtggccgaccctggttaccacc
 L  K  L  I  C  T  T  G  K  L  P  V  P  W  P  T  L  V  T  T
ctgggttatggtctgaaatgttttgcacgttatccggatcatatgaaacagcacgatttt
 L  G  Y  G  L  K  C  F  A  R  Y  P  D  H  M  K  Q  H  D  F
ttcaaaagcgcaatgccggaaggttatgttcaagaacgtaccatctttttcaaagacgac
 F  K  S  A  M  P  E  G  Y  V  Q  E  R  T  I  F  F  K  D  D
ggcaactataaaacccgtgccgaagttaaatttgaaggtgataccctggtgaatcgcatt
 G  N  Y  K  T  R  A  E  V  K  F  E  G  D  T  L  V  N  R  I
gaactgaaaggtattggctttaaagaagatggcaatatcctgggccataaactggaatat
 E  L  K  G  I  G  F  K  E  D  G  N  I  L  G  H  K  L  E  Y
aatggtctggcaggtctgacccgtctgagctttgcaattctgaatccgaaatggcgtgaa
 N  G  L  A  G  L  T  R  L  S  F  A  I  L  N  P  K  W  R  E
gaaatgatgggtggcggccgcgatagtgctacccatattaaattctcaaaacgtgatgag
 E  M  M  G  G  G  R  D  S  A  T  H  I  K  F  S  K  R  D  E
gacggcaaagagttagctggtgcaactatggagttgcgtgattcatctggtaaaactatt
 D  G  K  E  L  A  G  A  T  M  E  L  R  D  S  S  G  K  T  I
agtacatggatttcagatggacaagtgaaagatttctacctgtatccaggaaaatataca
 S  T  W  I  S  D  G  Q  V  K  D  F  Y  L  Y  P  G  K  Y  T
tttgtcgaaaccgcagcaccagacggttatgaggtagcaactgctattacctttacagtt
 F  V  E  T  A  A  P  D  G  Y  E  V  A  T  A  I  T  F  T  V
aatgagcaaggtcaggttactgtaaatggctaa
 N  E  Q  G  Q  V  T  V  N  G  -
```

B pIVEX-CaBoFDH

```
atgtctggttctcatcatcatcatcatcatagcagcggccatatgaaaattgtgctggtg
 M  S  G  S  H  H  H  H  H  H  S  S  G  H  M  K  I  V  L  V
ctgtatgatgcaggtaaacatgcagcagatgaagaaaaactgtatggctgcaccgaaaat
 L  Y  D  A  G  K  H  A  A  D  E  E  K  L  Y  G  C  T  E  N
aaactgggtattgcaaattggctgaaagatcagggtcatgaactgattaccaccagtgat
 K  L  G  I  A  N  W  L  K  D  Q  G  H  E  L  I  T  T  S  D
aaagaaggtgaaaccagcgaactggataaacatattccggatgccgatattatcattacc
 K  E  G  E  T  S  E  L  D  K  H  I  P  D  A  D  I  I  I  T
accccgtttcatccggcatatatcaccaaagaacgtctggataaagccaaaaatctgaaa
 T  P  F  H  P  A  Y  I  T  K  E  R  L  D  K  A  K  N  L  K
ctggttgttgttgccggtgttggtagcgatcatattgatctggattatatcaatcagacc
 L  V  V  V  A  G  V  G  S  D  H  I  D  L  D  Y  I  N  Q  T
ggcaaaaaaatcagcgttctggaagttaccggtagcaatgttgttagcgttgcagaacat
 G  K  K  I  S  V  L  E  V  T  G  S  N  V  V  S  V  A  E  H
gttgttatgaccatgctggttctggttcgcaattttgttccggcacatgagcagattatt
 V  V  M  T  M  L  V  L  V  R  N  F  V  P  A  H  E  Q  I  I
aaccatgattgggaagttgcagccattgcaaaagatgcctatgatattgaaggtaaaacc
 N  H  D  W  E  V  A  A  I  A  K  D  A  Y  D  I  E  G  K  T
attgcaaccattggtgcaggtcgtattggttatcgtgttctggaacgtctgctgccgttt
```

I A T I G A G R I G Y R V L E R L L P F
aatccgaaagaactgctgtattatgattatcaggcactgccgaaagaagccgaagaaaaa
N P K E L L Y Y D Y Q A L P K E A E E K
gttggtgcccgtcgtgttgaaaatattgaagaactggttgcacaggccgatattgttacc
V G A R R V E N I E E L V A Q A D I V T
gttaatgcaccgctgcatgccggtacaaaaggtctgattaacaaagagctgctgagcaaa
V N A P L H A G T K G L I N K E L L S K
ttcaaaaaaggtgcatggctggttaataccgcacgtggtgcaatttgtgttgccgaagat
F K K G A W L V N T A **R** G A I C V A E D
gttgcagcagcactggaaagcggtcagctgcgtggttatggtggtgatgtttggtttccg
V A A A L E S G Q L R G Y G G D V W F P
cagccagcaccgaaagatcatccgtggcgtgatatgcgtaacaaatatggtgccggtaat
Q P A P K D H P W R D M R N K Y G A G N
gcaatgacaccgcattatagcggcaccaccctggatgcacagacccgttatgcagaaggc
A M T P H Y S G T T L D A Q T R Y A E G
accaaaaacattctggaaagcttttttcaccggcaaatttgattatcgtccgcaggatatt
T K N I L E S F F T G K F D Y R P Q D I
attctgctgaatggtgaatatgtgaccaaagcctatggcaaacatgataaaaaa
I L L N G E Y V T K A Y G K H D K K

C pET28a-CaBoFDH

atgggcagcagccatcatcatcatcatcacagcagcggcctggtgccgcgcggcagccat
M G S S H H H H H H S S G L V P R G S H
atgaaaattgtgctggtgctgtatgatgcaggtaaacatgcagcagatgaagaaaaactg
M K I V L V L Y D A G K H A A D E E K L
tatggctgcaccgaaaataaactgggtattgcaaattggctgaaagatcagggtcatgaa
Y G C T E N K L G I A N W L K D Q G H E
ctgattaccaccagtgataaagaaggtgaaaccagcgaactggataaacatattccggat
L I T T S D K E G E T S E L D K H I P D
gccgatattatcattaccaccccgtttcatccggcatatatcaccaaagaacgtctggat
A D I I I T T P F H P A Y I T K E R L D
aaagccaaaaatctgaaactggttgttgttgccggtgttggtagcgatcatattgatctg
K A K N L K L V V V A G V G S D H I D L
gattatatcaatcagaccggcaaaaaaatcagcgttctggaagttaccggtagcaatgtt
D Y I N Q T G K K I S V L E V T G S N V
gttagcgttgcagaacatgttgttatgaccatgctggttctggttcgcaattttgttccg
V S V A E H V V M T M L V L V R N F V P
gcacatgagcagattattaaccatgattgggaagttgcagccattgcaaaagatgcctat
A H E Q I I N H D W E V A A I A K D A Y
gatattgaaggtaaaaccattgcaaccattggtgcaggtcgtattggttatcgtgttctg
D I E G K T I A T I G A G R I G Y R V L
gaacgtctgctgccgtttaatccgaaagaactgctgtattatgattatcaggcactgccg
E R L L P F N P K E L L Y Y D Y Q A L P
aaagaagccgaagaaaaagttggtgcccgtcgtgttgaaaatattgaagaactggttgca
K E A E E K V G A R R V E N I E E L V A
caggccgatattgttaccgttaatgcaccgctgcatgccggtacaaaaggtctgattaac
Q A D I V T V N A P L H A G T K G L I N
aaagagctgctgagcaaattcaaaaaaggtgcatggctggttaataccgcacgtggtgca
K E L L S K F K K G A W L V N T A R G A
atttgtgttgccgaagatgttgcagcagcactggaaagcggtcagctgcgtggttatggt
I C V A E D V A A A L E S G Q L R G Y G
ggtgatgtttggtttccgcagccagcaccgaaagatcatccgtggcgtgatatgcgtaac
G D V W F P Q P A P K D H P W R D M R N
aaatatggtgccggtaatgcaatgacaccgcattatagcggcaccaccctggatgcacag
K Y G A G N A M T P H Y S G T T L D A Q
acccgttatgcagaaggcaccaaaaacattctggaaagcttttttcaccggcaaatttgat
T R Y A E G T K N I L E S F F T G K F D
tatcgtccgcaggatattattctgctgaatggtgaatatgtgaccaaagcctatggcaaa
Y R P Q D I I L L N G E Y V T K A Y G K
catgataaaaaaggcggtggtagtgcacatattgttatggttgatgcatacaaaccgacc
H D K K G G G S A H I V M V D A Y K P T

aaaggtagcggtagcggccgctatccgtatgatgtaccagattatgcaagcctctaa
K G S G S G R Y P Y D V P D Y A S L -

D pET28a-CaBoFDH_BsaI*2 (GoldenGate acceptor vector)

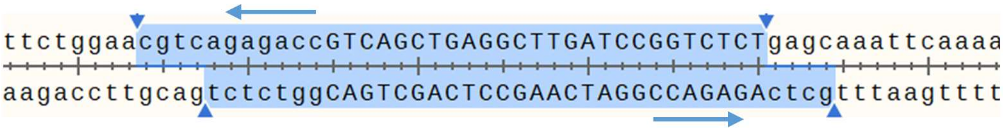

Figure S1: Partial DNA and amino acid sequences of constructs used in this study . (A) Open reading frame for pET28a-SoNar-SpyCatcher. The polyHistag is highlighted in green, the SoNar sequence in yellow and the SpyCatcher sequence in red. (B) Open reading frame for pIVEX-CaBoFDH construct. The polyHistag is highlighted in green, the CaBoFDH sequence in grey. Position R258 (mutated to R258A to create inactive mutant) is underlined. (C) Open reading frame for the pET28a-CaBoFDH construct. The polyHistag is highlighted in green, the CaBoFDH sequence in grey. (D) Partial DNA sequence for pET28a-CaBoFDH_BsaI*2 (GoldenGate acceptor vector) used for recovery of DNA from sorted beads, showing the orientation of Eco31I sites (blue arrows) and the insert (DNA sequence highlighted in blue) that was removed during the GoldenGate procedure.

Table S1. Oligonucleotides used in this study for non-SpliMLiB cloning

Name	Sequence 5'->3'
FREXtoPOP_F	TAATTGACTAGTGGATCCATGAATAAAGACCAGAGCAAAATTC
FREXtoPOP_R	TTCTTAGCGGCCGCTTAACTACCACCCAGTTTTTCGATTTCTTCCAG
SpyC_pETTerm_F	ctgtaaatggctaaGCTAACAAAGCCCGAAAGGAAGCTG
Thrombin_SoNar_R	CGATTACCGCTGCCGCGCGGCAC
Thrombin_SoNar_F	CGCGGCAGCGGTAATCGTAAATGGGGTCTGTGTATTGTT
SoNar_NotI_SpyC_R	atcGCGGCCGCCACCCATCATTTCTTCACGCCATTTC
SoNar_NotI_SpyC_F	GTGGCGGCCGCgatagtgctacccatattaaattctcaaaacgtg
SpyC_pETTerm_R	CGGGCTTTGTTAGCttagccatttacagtaacctgaccttgctc
R258A_F	CGCAGCGGGTGCAATTTGTGTTGCCGAAG
R258A_R	GCACCCGCTGCGGTATTAACCAGCCATGC
BsaI_Lib_F	gtattgGGTCTCTCGTCTGCTGCCGTTTAATCCGAAAGAACTGCTGTAT
BsaI_Lib_R	gttatgggtctctGCTCAGCAGCTCTTTGTTAATCAGACCTTTTGT
BsaI_Vec_SDM_F	GTCAGCTGAGGCTTGATCCGGTCTCTGAGCAAATTCAAAAAAGGTGCATGGCTGGTTAATACCGCAC
BsaI_Vec_SDM_R	GGATCAAGCCTCAGCTGACggtctctGACGTTCCAGAACACGATAACCAATACGACCTGCACC

Table S2. Common oligonucleotides used to generate DNA for SpliMLiB**.**

Oligonucleotide	5'-modification	Sequence (5'->3')
T7t-DBCO	DBCO	gctagttattgctcagcgg
LMB-Cy5	Cy5	atgtgctgcaaggcgattaag
LMB-TxsRed	TexasRed	atgtgctgcaaggcgattaag

Table S3. Oligonucleotides for construction of the CaBoFDH SpliMLiB library. All oligonucleotides below were from SigmaAldrich (Haverhill, England) and were synthesized without any 5'-OH modification. The BspQI recognition site (GCTCTTC) is in purple, the Eco31I recognition sequence (CGTCTC) is in red, the 5'-single stranded overhangs that resulted from Esp3I digestion (for T10X_R and M35X_F oligonucleotides) or that were introduced by design (M18X_F & M18X-R, G28X_F & G28X_R oligonucleotides) is in blue and the mutant codon is in green. Each series consisted of 20 different oligonucleotides, varying at the position marked 'XXX', containing the site saturation codons listed in Table S4.

Oligonucleotide series	Sequence (5'->3')
Y194X_R	gttatgGCTCTTCAGTCXXXATACAGCAGTTCTTTCGGATTAAACGGC
Y196X_F	GGATCCGGTGGCAAGCTGGAGGTGCTGCTCTTCAGACXXX
Y196X_R	CCTGXXXGTCTGAAGAGCAGCACCTCCAGCTTGCCACCGGATCC
A229_F	gtattgCGTCTCTCAGGCACTGCCGAAAGAAGCC
A229X_R	gttatgCGTCTCTGCAGCGGXXXATTAACGGTAACAATATCGGCCTGTGC
G234X_F	gtattgCGTCTCTCTGCATGCCXXXACAAAAGGTCTGATTAACAAAGAGCTGCTG

Table S4. Codons for site saturation in the SpliMLiB library. The DNA sequence shown in this table was used at the position of the green 'XXX' in Table S3 above, for the oligonucleotide series Y196X_F and G234X_F. For the oligonucleotide series Y194X_R and Y196X_R, the reverse complement of the DNA sequence in this table was used at the position of the green 'XXX' in Table S3.

amino acid	codon	amino acid	codon
A	GCT	M	ATG
C	TGT	N	AAT
D	GAT	P	CCG
E	GAA	Q	CAA
F	TTT	R	CGT
G	GGT	S	TCT
H	CAT	T	ACC
I	ATC	V	GTG
K	AAA	W	TGG
L	CTG	Y	TAT

Table S5: Amino acids targeted at each position

194	196	229	234
		A	A
C	C	C	C
	D	D	D
			E
F	F		
		G	G
H	H		
I		I	
	K		K
	L		
M		M	
N	N		N
P		P	P
Q	Q		Q
	R	R	R
	S	S	S
T		T	T
V		V	
W	W		
Y	Y	Y	

Table S6 PCR amplicons used in this study.

PCR product name	Forward primer	Reverse primer	Template
Cy5-WT-FDH	Cy5-LMB	T7t-DBCO	pIVEX-CaBoFDH
TxsRed-R258A-FDH	TxsRed-LMB	T7t-DBCO	pIVEX-CaBoFDH R258A
frag$_{G234}$	G234X_F	T7t-DBCO	pIVEX-CaBoFDH
frag$_{A229}$	A229_F	A229X_R	pIVEX-CaBoFDH
frag$_{Y194}$	LMB-Cy5	Y194X_R	pIVEX-CaBoFDH

Table S7. Oligonucleotide duplex used for CaBoFDH SpliMLiB library generation

ON duplex	ON 1	ON 2
frag$_{Y196}$	Y196X_F	Y196X_R

Step	Bead or off-bead operation		Split or mix
1	off-bead	***frag$_{G234}$ production (by PCR)***<br>5'-GTATTGCGTCTCACTGCCATGCCXXXACAAAAG…-3'<br>3'-CATAACGCAGAGTGACGGTACGGXXXTGTTTTC…-5'-DBCO	split
2	bead	***immobilization of frag$_{G234}$ (by click reaction)***<br>5'-GTATTGCGTCTCACTGCCATGCCXXXACAAAAG…-3'<br>3'-CATAACGCAGAGTGACGGTACGGXXXTGTTTTC…-5'-DBCO--------> bead	split
3	bead	***Esp3I digestion***<br>P-5'-CTGCCATGCCXXXACAAAAG…-3'<br>3'-GTACGGXXXTGTTTTC…-5'-DBCO---------bead	mix
4	off-bead	***frag$_{A229}$ production (by PCR)***<br>5'-GTATTGCGTCTCTCAGGCACTGC[…]TTAATXXXCCGCTGCAGAGACGCATAAC-3'<br>3'-CATAACGCAGAGAGTCCGTGACG[…]AATTAXXXGGCGACGTCTCTGCGTATTG-5'	split
5	off-bead	***Esp3I digestion***<br>P-5'-CAGGCACTGC[…]TTAATXXXCCG-3'<br>GTGACG[…]AATTAXXXGGCGACG-5'-P	split
6	bead	***ligation of digested frag$_{A229}$***<br>P-5'-CAGGCACTGC[…]TTAATXXXCCGCTGCCATGCCXXXACAAAAG…-3'<br>3'-GTGACG[…]AATTAXXXGGCGACGGTACGGXXXTGTTTTC…-5'-DBCO---------bead	split
7	bead	***Esp3I digestion (to ensure complete liberation of sticky end)***<br>P-5'-CAGGCACTGC[…]TTAATXXXCCGCTGCCATGCCXXXACAAAAG…-3'<br>3'-GTGACG[…]AATTAXXXGGCGACGGTACGGXXXTGTTTTC…-5'-DBCO---------bead	mix
8	off-bead	***frag$_{Y196}$ production (by oligonucleotide hybridization)***<br>5'-GGATCCGGTGGCAAGCTGGAGGTGCTGCTCTTCAGACXXX-3'<br>3'-CCTAGGCCACCGTTCGACCTCCACGACGAGAAGTCTGXXXGTCC-5'-P	split
9	bead	***ligation of frag$_{Y196}$***<br>5'-GGATCCGGTGGCAAGCTGGAGGTGCTGCTCTTCAGACXXXCAGGCACTGC[…]TTAATXXXCCGCTGCCATGCCXXXACAAAAG…-3'<br>3'-CCTAGGCCACCGTTCGACCTCCACGACGAGAAGTCTGXXXGTCCGTGACG[…]AATTAXXXGGCGACGGTACGGXXXTGTTTTC…-5'-DBCO---------bead	split
10	bead	***BspQI digestion***<br>P-5'-GACXXXCAGGCACTGC[…]TTAATXXXCCGCTGCCATGCCXXXACAAAAG…-3'<br>3'-XXXGTCCGTGACG[…]AATTAXXXGGCGACGGTACGGXXXTGTTTTC…-5'-DBCO---------bead	mix
11	off-bead	***frag$_{Y194}$ production (by PCR)***<br>Cy5-5'-…TGTATXXXGACTGAAGAGCCATAAC-3'<br>3'-…ACATAXXXCTGACTTCTCGGTATTG-5'	split
12	off-bead	***BspQI digestion***<br>Cy5-5'-…TGTATXXX-3'<br>3'-…ACATAXXXCTG-5'-P	split
13	bead	***ligation of frag$_{Y194}$***<br>Cy5-5'-TGTATXXXGACXXXCAGGCACTGC[…]TTAATXXXCCGCTGCCATGCCXXXACAAAAG…-3'<br>3'-ACATAXXXCTGXXXGTCCGTGACG[…]AATTAXXXGGCGACGGTACGGXXXTGTTTTC…-5'-DBCO---------bead	split

Figure S2. Sequence-level overview of fragment preparation and solid-phase manipulation (immobilisation, digestion & ligation) of DNA for the CaBoFDH SpliMLiB library generation. In step 1, frag$_{G234}$ (**Table S6**) was produced by PCR in 12 separate iterations (i.e. in 'split' mode), these PCR fragments, carrying a DBCO functionality at one of their 5'-ends, were immobilized onto the azide-functionalized beads in step 2, in split-mode (12 separate immobilizations for each fragment, ensuring bead 'monoclonality'). Beads were then mixed into a single pot and their immobilized DNA was digested by Esp3I (step 3). Separately, frag$_{A229}$ (**Table S6**) was prepared in split-mode by PCR (step 4), subjected to Esp3I digestion in split-mode (step 5) and ligated to the DNA on beads; aliquots of beads (from step 3) had been distributed across the different splits (step 6). Subsequently, beads were mixed and subjected to a further digestion with Esp3I (step 7), to ensure complete liberation of the single stranded 5'-overhang at the 'growing' end of the DNA (i.e., the non-immobilized 5'-end). Separately, frag$_{Y196}$ (**Table S7**) was prepared in split-mode through annealing of sets of two partially complementary oligonucleotides, which were ligated to bead-immobilized DNA, again in split reactions (step 9). After mixing the beads, a 3-base, single stranded, 5'-overhang was generated by treating the bead immobilized DNA with BspQI (step 10). The final set of fragments, frag$_{Y194}$, was prepared by PCR (**Table S6**) in split-mode (step 11) and subjected to BspQI digestion (step 12), before the fragments were ligated to bead-immobilized DNA in separate, split reactions (step 13). The beads from step 13, once mixed, represented the final CaBoFDH SpliMLiB library.

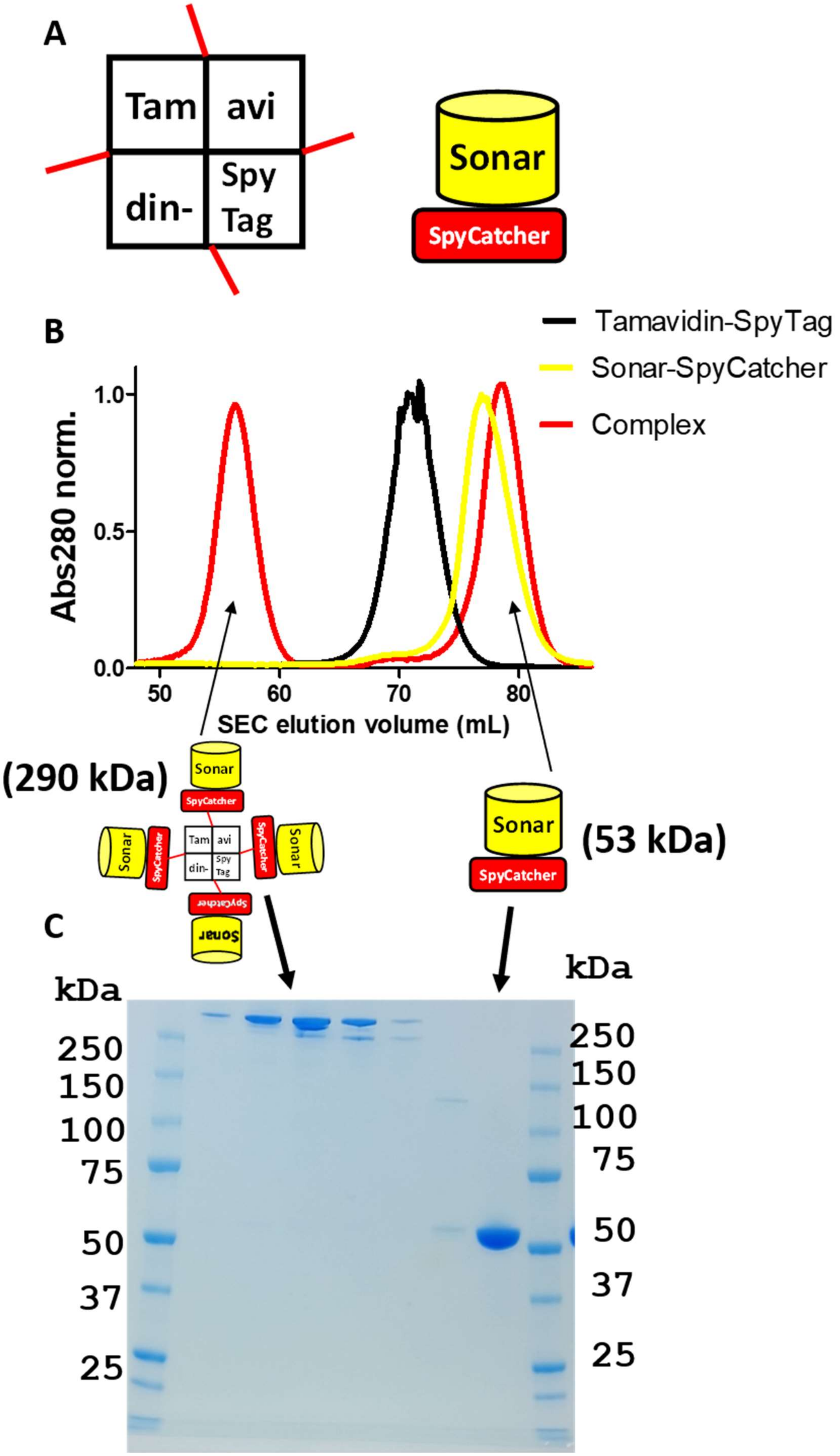
A
Tam
avi
din-
Spy Tag
Sonar
SpyCatcher
B
Tamavidin-SpyTag
Sonar-SpyCatcher
Complex
Abs280 norm.
1.0
0.5
0.0
50
60
70
80
SEC elution volume (mL)
(290 kDa)
(53 kDa)
C
kDa
250
150
100
75
50
37
25

Figure S3: Preparation of post-translationally fused protein complex from Tamavidin-2-HOT-SpyTag and SoNar-SpyCatcher. (A) Schematic representation of the recombinant proteins Tamavidin-2-HOT-SpyTag (a tetramer) and SoNar-SpyCatcher (a monomer). (B) Size exclusion elution profile for the two proteins, in isolation, or after complex formation. (C) SDS PAGE analysis of SEC (sample labelled “Complex” in panel B). Numbers at the top of the gel indicate SEC elution volume in mL, as in panel B.

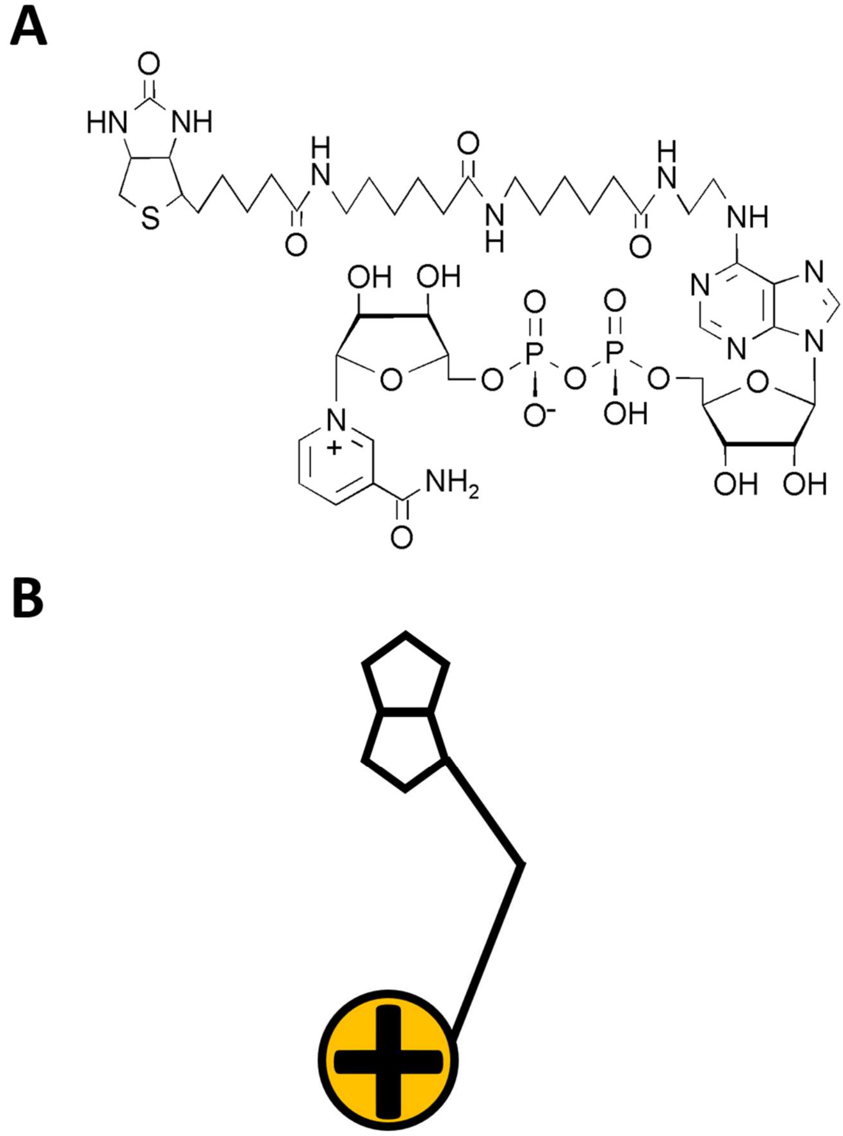

Figure S4: Biotin-17-NAD structure. (A) Chemical structure of a commercially available analogue of NAD+ (biotin-17-NAD+) in which the exocyclic nitrogen of the adenine moiety is functionalized with a linker and biotin (obtained from Biolog Life Science Institute GmbH & Co. KG). (B) The schematic depiction which we use for biotin-17-NAD in Figures 1, 2 & 3. The “+” symbol in the yellow circle represents NAD^+, while a “H” was used in this yellow circle to represent NADH in Figure 1 (iv), Figure 2B & 2C and Figure 3A.

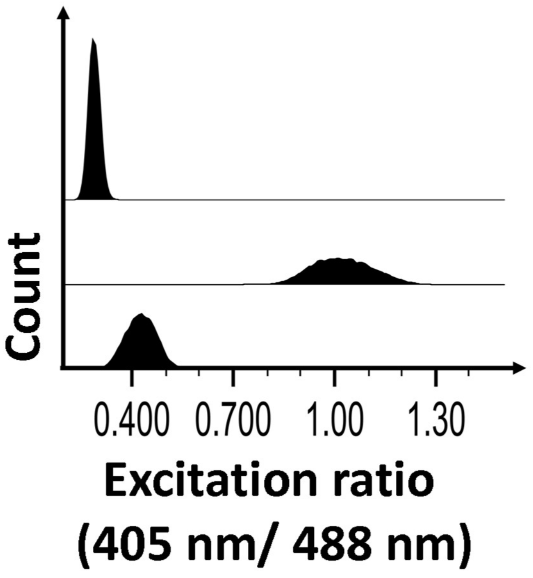

Figure S5: Demonstration of background oxidation activity in IVTT mixture. Beads with immobilized NAD+ were left untreated (top trace), exposed to sodium lactate & LDH (middle trace), or exposed to the latter, washed and then exposed to IVTT for 6 hours at 25 °C (bottom trace).

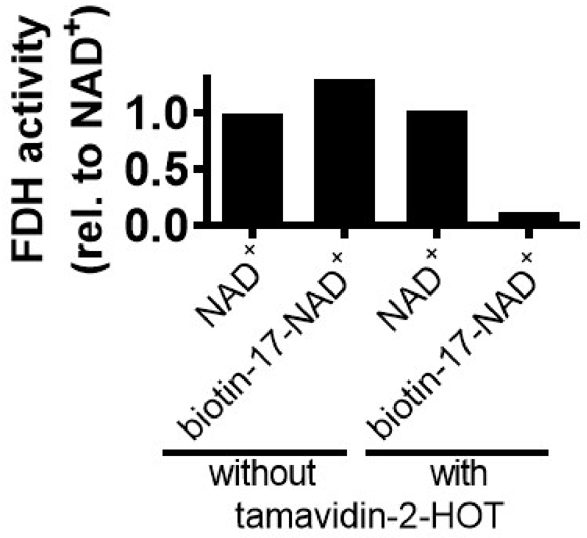

Figure S6: Activity of CaBoFDH with biotin-17-NAD+ substrate in the absence or presence of Tamavidin-2-HOT. The enzyme concentration was 0.1 µM, the NAD+ or biotin-17-NAD+ concentrations were 10 µM, the concentration of sodium formate was 25 mM and the buffer consisted of 50 mM Tris-Cl (pH 8) and 100 mM NaCl. Activity was normalized to activity observed with NAD+ in absence of Tamavidin-2-HOT.

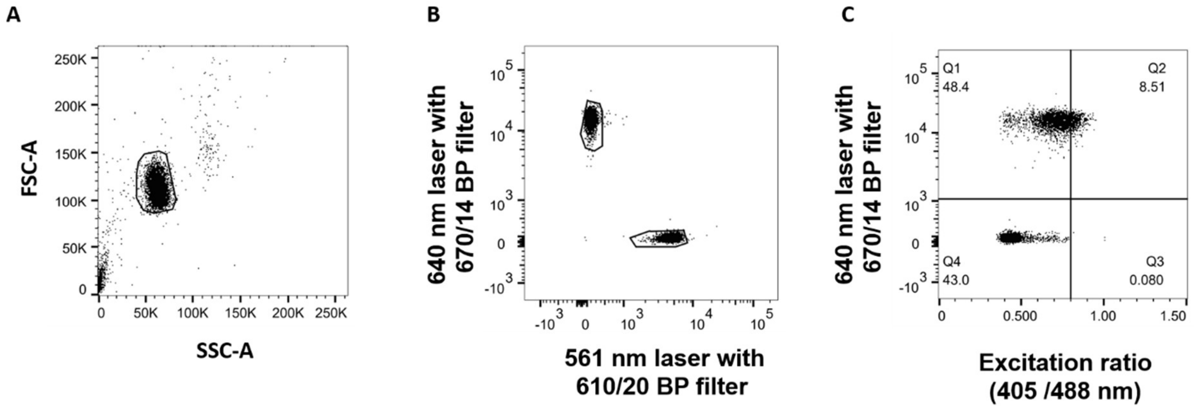

Figure S7: Flow cytometry gating scheme and NAD-display assay's positive predictive value. All three graphs are based on the same data, taken from sample depicted in Figure 3B (iv). (A) Forward scatter vs side scatter dot plot with showing the gate applied to select for single beads. (B) The subpopulation from panel (A) was further gated for either Cy5 fluorescence (high intensity fluorescence from the 640 nm laser with 670 nm emission) or TexasRed fluorescence (high intensity fluorescence from the 561 nm laser with 610 nm emission). (C) Dotplot setting out Cy5 fluorescence emission against the 405/488 nm SoNar excitation ratio. The plot was divided into four quadrants. The Y-axis-intersecting line divides the readily identifiable genotypes (wildtype-Cy5 and inactive mutant-TexasRed), while the X-axis-intersecting line is based on the lower limit of the theoretical sorting range depicted in Figure **S7**3. The four quadrants thus represent the false negatives (Q1), the true positives (Q2), the false positives (Q3) and the true negatives (Q4).

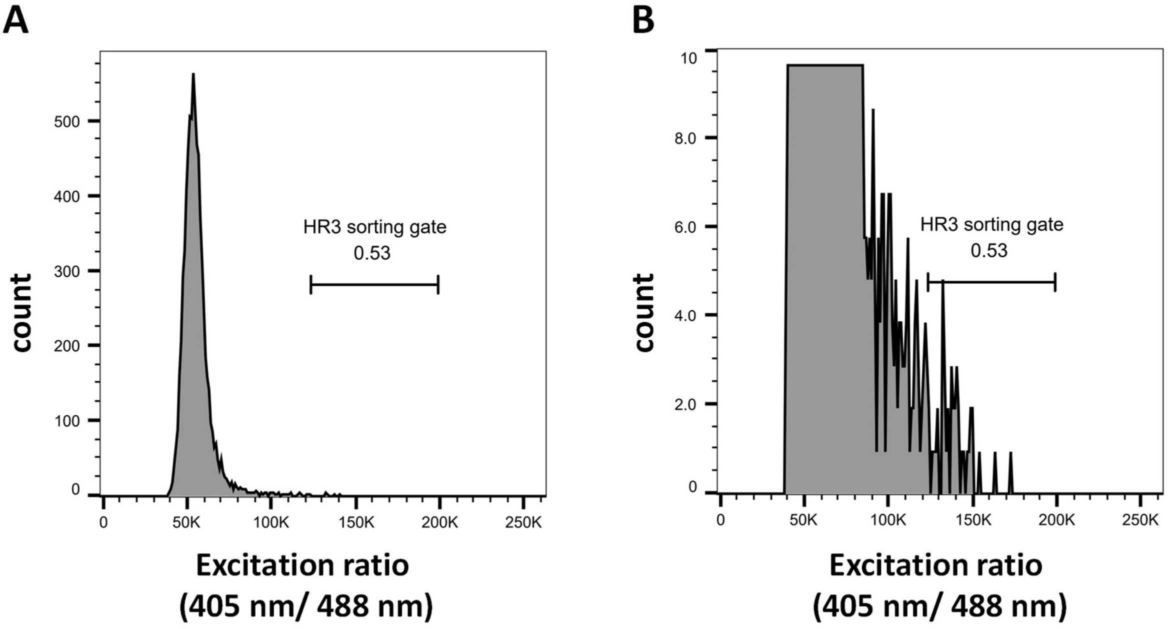

Figure S8: FACS sorting histogram showing the bead count as function of the SoNar fluorescence excitation ratio (405 nm/488 nm). A total of 237,000 events were registered by the FACS, while 215 beads were sorted by gate HR3, were taken for further analysis in the secondary, bacterial lysate-based assay (Figure 4B). The full data set is shown in (A), while the Y-scale is reduced in (B) to show the relatively rare events that were sorted at the tail-end of the distribution.

Table S8: CaBoFDH SpliMLiB input library clones' activity (normalized to wildtype) and sequences. 92 clones were assessed for activity and the first 36 of these were also sequenced.

clone	activity	Y194	Y196	A229	G234	Notes
1	0.12	W	Y	M	A	
2	0.13	F	H	V	R	
3	0.01	N	Y	Y	P	
4	0.08	W	C	S	C	
5	-0.01	Y	C	M	Q	
6	0.16	H	S	M	R	
7	0.03	Q	H	P	E	
8	0.02	F	H	G	E	
9	0.20	I	W	C	R	
10	0.03	V	L	A	T	
11	0.18	H	S	M	R	
12	0.05	H	L	T	P	
13	0.06	Y	H	A	T	
14	0.12	Y	L	M	E	
15	0.07	H	Q	V	T	
16	0.02	N	D	S	R	
17	0.14	H	H	C	D	
18	0.19	F	W	V	C	
19	0.02	P	W	A	S	
20	0.03	V	L	A	E	
21	0.01	I	Q	R	N	
22	0.02	V	L	A	R	
23	0.02					deletion
24	-0.06					deletion
25	0.04	H	S	P	G	
26	0.01	M	R	S	P	
27	0.07	N	N	I	C	
28	0.13	C	N	A	C	
29	0.06					double peaks in chromatogram
30	0.01	P	N	G	A	
31	0.89	Y	Y	C	Q	
32	0.01	T	L	Y	C	
33	0.04	T	R	I	N	
34	1.12	Y	Y	A	G	WT contaminant
35	0.02	P	Y	P	C	
36	0.93	Y	Y	C	Q	
37	0.05					
38	0.02					
39	0.10					
40	0.01					
41	0.13					
42	0.13					
43	0.06					
44	0.04					
45	0.10					

46	0.17	
47	0.12	
48	0.15	
49	0.00	
50	0.10	
51	0.02	
52	0.01	
53	0.02	
54	0.04	
55	0.03	
56	0.15	
57	0.04	
58	0.02	
59	0.03	
60	0.03	
61	0.01	
62	0.04	
63	0.03	
64	0.02	
65	0.06	
66	0.04	
67	0.04	
68	0.01	
69	0.07	
70	0.21	
71	0.03	
72	0.02	
73	0.01	
74	0.02	
75	0.05	
76	0.31	
77	0.13	
78	0.02	
79	0.08	
80	0.02	
81	0.26	
82	0.36	
83	0.04	
84	0.04	
85	0.02	
86	0.03	
87	0.03	
88	0.02	
89	0.29	
90	0.04	
91	0.10	
92	0.00	

Table S9: CaBoFDH SpliMLiB NAD-display screened clones' activity (normalized to wildtype) and sequences. 92 clones were assessed for activity and the first 36 of these were also sequenced.

clone	activity	Y194	Y196	A229	G234	Notes
1	0.26	F	Y	V	N	
2	-0.01	F	L	T	Q	deletion
3	0.86	I	Y	C	Q	
4	0.98	F	Y	A	G	
5	0.91	Y	Y	C	K	
6	0.94	F	Y	T	G	
7	0.81	I	Y	C	D	
8	0.86	Y	Y	V	G	
9	0.17	F	F	V	K	
10	0.70	Y	Y	C	T	
11	0.89	C	Y	C	K	
12	0.50	Y	Y	V	D	
13	0.60	F	Y	C	T	
14	0.87	Y	Y	C	D	
15	0.69	C	Y	C	T	
16	0.84	Y	Y	C	D	
17	0.24	H	Y	S	S	
18	0.82	F	Y	T	G	
19	0.48	Y	Y	V	D	
20	0.07	F	S	C	T	
21	0.42	H	Y	A	D	
22	0.82	F	Y	C	K	
23	0.72	C	Y	C	T	
24	0.77	Y	Y	C	Q	
25	0.91	F	Y	A	G	
26	0.61	Y	Y	T	K	
27	1.02	F	Y	C	G	
28	0.02	F	del	R	D	deletion
29	0.33	T	Y	A	S	
30	0.30	I	Y	C	C	
31	0.21	M	Y	C	C	
32	0.54	Y	Y	V	K	
33	0.23	F	Y	C	C	
34	0.10	del	del	C	Q	deletion
35	0.12	H	Y	C	R	
36	0.85	Y	Y	C	Q	
37	0.78					
38	0.76					
39	0.17					
40	0.48					
41	0.49					
42	0.79					
43	0.76					
44	0.57					
45	0.66					

46	0.79	
47	0.77	
48	0.86	
49	-0.01	
50	0.01	
51	0.50	
52	0.78	
53	0.76	
54	0.83	
55	0.84	
56	0.81	
57	0.91	
58	-0.09	
59	0.27	
60	0.73	
61	0.56	
62	0.56	
63	0.29	
64	0.69	
65	0.68	
66	0.73	
67	0.02	
68	0.09	
69	0.71	
70	0.36	
71	0.36	
72	0.11	
73	0.70	
74	0.40	
75	0.01	
76	0.69	
77	0.73	
78	0.58	
79	0.91	
80	0.72	
81	0.74	
82	0.75	
83	0.70	
84	0.53	
85	0.87	
86	0.59	
87	0.81	
88	0.02	
89	0.66	
90	0.62	
91	0.68	
92	0.77	

References

[1] Y. P. Hung, J. G. Albeck, M. Tantama, G. Yellen, *Cell Metab.* **2011**, *14*, 545–54.

[2] J. Peränen, M. Rikkonen, M. Hyvönen, L. Kääriäinen, *Anal. Biochem.* **1996**, *236*, 371–373.

[3] J. Quan, J. Tian, *PLoS One* **2009**, *4*, e6441.

[4] L. Lindenburg, T. Huovinen, K. van de Wiel, M. Herger, M. R. Snaith, F. Hollfelder, *Nucleic Acids Res.* **2020**, DOI 10.1093/nar/gkaa270.

[5] H. Liu, J. H. Naismith, *BMC Biotechnol* **2008**, *8*, 91.

[6] H. Wang, H. Ceylan Koydemir, Y. Qiu, B. Bai, Y. Zhang, Y. Jin, S. Tok, E. C. Yilmaz, E. Gumustekin, Y. Rivenson, et al., *Light Sci. Appl.* **2020**, *9*, DOI 10.1038/s41377-020-00358-9.

[7] M. F. Debets, S. S. van Berkel, J. Dommerholt, A. (Ton) J. Dirks, F. P. J. T. Rutjes, F. L. van Delft, *Acc. Chem. Res.* **2011**, *44*, 805–815.

[8] L. Diamante, P. Gatti-Lafranconi, Y. Schaerli, F. Hollfelder, *Protein Eng. Des. Sel.* **2013**, *26*, 713–24.

[9] M. Fischlechner, Y. Schaerli, M. F. Mohamed, S. Patil, C. Abell, F. Hollfelder, *Nat Chem* **2014**, *6*, 791–796.

[10] C. Engler, R. Kandzia, S. Marillonnet, *PLoS One* **2008**, *3*, e3647.
